# Salmagundi

**Published:** 1885-11

**Authors:** 


### Salmagundi.

EDITED BY E. G. BETTY, D. D. S.

Dammar Cement.—Dissolve gum dammar in benzole, and one-third of gold-size; it dries very quickly, and is preferably used as a first coat for affixing the cover-glass when glycerine is used for mounting.—*The Microscope.*

After carefully and laboriously measuring many hundred red corpuscles of human blood, Dr. Marshall D. Ewell finds the average size to be 1-3162 of an inch, the smallest of the whole number was 1-5050, the largest 1-2544 of an inch.

"NEARLY a half in number and two-thirds in circulation of the newspapers of the world are printed in English." The same at least, or greater, would about represent the dental periodicals of the world, only with the difference that the big end of the list is published in the United States.

---

THOSE of our readers who are interested in microscopical work will find the slide made by the Palmer Slide Co., of Geneva, N. Y., to be the most beautiful and perfect thing of the kind; the edges are beveled and polished, giving the slide a unique beauty that adds greatly to the appearance of finished work.

---

M. PAUL VIGIER recommends (*Gazette Hebdomadaire*, July 24, 1885,) what he terms a *sirop de dentition* to assuage the pain suffered by infants while cutting their teeth, especially the canines; hydrochlorate of cocaine, 10 centigrammes; simple syrup, 10 grammes; tincture of saffron, 10 drops. The painful gums should be gently rubbed with some of this several times a day.

---

"DR. LANDER BRUNTON said recently that headaches were usually dependent either upon the presence of decayed teeth, or of some irregularity in the eyes, more especially in the focal lengths between the two. Another irregularity of the eyes that has a bearing on the case, is that which is caused by compelling them to look too often through the bottoms of certain styles of glasses."

---

G. BUNGE says he is led to believe that food does not contain iron in inorganic combinations, but that it only exists in complex organic compounds which have been formed by the vital processes. It is absorbed and assimilated in this form and then converted into hæmoglobin."

It would appear from this that iron cannot enter directly into the organic system without having previously passed through the vegetable.

Dr. Combs states that there is a peculiar form of caries which attacks the teeth of those addicted to the use of morphine. It appears first on the triturating surface of the large molars, and extends rapidly until a large cavity is formed. The bicuspids, incisors, and canines are then attacked in succession. This form of caries is nearly painless, advances with great rapidity, and is unaccompanied by periostitis. The dental lesions occur usually at the same time with the falling out of the hair.

---

"M. Gellette overcomes the odor of iodoform by mixing it with charcoal and sulphate of quinine in the following proportions:

| | | |
|---|---|---|
| Iodoform | 100 | grammes. |
| Sulphate of Quinine | 1 | gramme. |
| Charcoal | 3 | grammes. |

This recipe will be a valuable one to the dentist, for next to a stinking mouth, iodoform is one of the most intensely disagreeable odors he is obliged to inhale.

---

### SALICYLATE OF COCAINE.

Recently a new remedy has been brought forward, which promises to become the specific in neuralgia of the trigeminus. Dr. M. Schneider (*Allg. Med. Centr. Zeit.*, 49, 1885,) had a lady patient, who at long intervals suffered from severe attacks of this painful malady. These attacks lasted from one month to half a year, and were accompanied by the most excruciating pains. Five years ago such an attack had been stopped by large doses of quinine. Another attack, which had been continued with little interruption for six months, finally yielded to morphia and iron. A month ago the patient was again attacked by her old enemy. None of the remedies formerly applied seemed to do any good. S. then employed as an experiment salicylate of cocaine. Two-thirds of a grain were injected hypodermically into the right cheek. A minute or two later the pain ceased, and did not return. The injection itself was painless, and produced no irritation.

A week ago Engel had a similar case. The patient, a young man, presented himself at the clinic of Prof. Engel in Medico-Chirurgical College. He suffered excruciating pains, and near the left ala nasi was a very tender spot. Thirty minims of a two per cent. solution of muriate of cocaine were injected into the skin near the point douloureux. Scarcely a minute later the pain had ceased. The remedy surely merits a more extensive trial.—*Medical and Surgical Reporter.*

---

FOR DIARRHŒA DURING DENTITION.

EDITOR MEDICAL WORLD:

I send you the following prescription for diarrhœa during dentition. The child's second summer with many, is a critical period of existence. Whether the actions be chylous or mucous, or whether there be hepatic derangement, it seems to meet every indication and has been quite successful in my hands.

| | | |
|---|---|---|
| ℞ | Plumbi acetat................................ | gr. x |
| | Cret. prep.................................... | ℥ ss |
| | Hydrarg. chlor. mit........................ | gr. v |
| | Pulv. Dov.................................... | gr. x |

M.—Ft. pulv. et. chart. x. One every two or four hours.

This prescription might seem unscientific owing to the incompatibility between calomel and lead. Be that as it may, if new compounds are formed by the interchange of elements, I know the combination to be harmless, and it results in great good.

GEORGE W. BOWLEN, M.D.

BARNESVILLE, MD.

---

DENTISTS'S LEG.—A peculiar sensation of numbness, or "pins and needles," in the extremities is frequently experienced by dentists. This condition Dr. George Johnson (*Lancet*, August 15,) considers to be due to the combined influence of perverted nerve function, directly due to a mechanical impediment to the circulation through the rigidly contracted muscles and their associated nerves, and to direct compression of the nerves by the firmly contracted muscles. This combination is found in dentists who stand fixed and firm in one position for long periods of time. The obvious means of prevention and of cure consist in rest for the over-strained limb, or such a frequent change of position as is equivalent to a certain amount of rest. Standing in one position is notoriously more fatiguing than walking, and for the obvious reason that while in standing one set of muscles is in a constant state of active contraction, the circulation through them being thereby retarded and enfeebled, walking involves alternate contraction and relaxation of the muscles, with an invigorated and quickened circulation.

